# Recognizing the hidden: strengthening the HIV surveillance system among key and priority populations in Mozambique

**DOI:** 10.1186/s12889-020-10110-y

**Published:** 2021-01-07

**Authors:** Cynthia Semá Baltazar, Makini Boothe, Denise Chitsondzo Langa, Isabel Sathane, Roberta Horth, Peter Young, Nick Schaad, Henry F. Raymond

**Affiliations:** 1 Instituto Nacional de Saúde (INS), Maputo, Mozambique; 2 Department of Public Health and Primary Care, Faculty of Medicine and Health Sciences, Ghent University, Ghent, Belgium; 3 HIV and STI Program, Public Health Directorate, Ministry of Health, Maputo, Mozambique; 4 Centers for Disease Control and Prevention, Atlanta, USA; 5 Centers for Disease Control and Prevention, Maputo, Mozambique; 6 University of California, San Francisco (UCSF), San Francisco, USA; 7 School of Public Health, Rutgers University, Piscataway, NJ USA

**Keywords:** HIV, Surveillance, Biological and behavioral surveys, Mozambique, Key population, Priority population, Lessons learned

## Abstract

High quality, representative data from HIV surveillance systems that have country ownership and commitment are critical for guiding national HIV responses, especially among key and priority populations given their disproportionate role in the transmission of the virus. Between 2011 to 2013, the Mozambique Ministry of Health has conducted five Biobehavioral Surveillance Surveys among key populations (female sex workers, men who has sex with men and people who inject drugs) and priority populations (long distance truck drives and miners) as part of the national HIV surveillance system. We describe the experience of strengthening the HIV surveillance system among those populations through the implementation of these surveys in Mozambique. We document the lessons learned through the impact on coordination and collaboration; workforce development and institutional capacity building; data use and dissemination; advocacy and policy impact; financial sustainability and community impact. Key lessons learned include the importance of multisectoral collaboration, vital role of data to support key populations visibility and advocacy efforts, and institutional capacity building of government agencies and key populations organizations. Given that traditional surveillance methodologies from routine data often do not capture these hidden populations, it will be important to ensure that Biobehavioral Surveillance Surveys are an integral part of ongoing HIV surveillance activities in Mozambique.

## Background

Mozambique has a generalized HIV epidemic with a prevalence of 13.2% among adults aged 15–49 years old [1]. Despite the implementation of activities to prevent and control the epidemic, HIV remains one of the greatest public health challenges in the country. Early in the HIV epidemic in Mozambique, like elsewhere in sub-Saharan Africa, disease monitoring relied on sentinel surveillance in priority groups, such as pregnant women, and later nationally representative population-based household surveys [1, 2]. A limitation of population-based household surveys is that they are often unable to sufficiently sample numbers of vulnerable populations such as migrants and mobile populations, displaced persons, or key populations (KP), such as sex workers, men who have sex with men, people who inject drugs, transgender people, and their sexual partners, who disproportionately contribute to HIV transmission due to high risk sexual and drug use behaviors [3–5]. Additionally, because of stigma and discrimination, individuals in high-risk populations may not self-identify as such in household-based surveys. Therefore, global agencies such as the Joint United Nations Programme on HIV/AIDS (UNAIDS), World Health Organization (WHO), President’s Emergency Plan for AIDS Relief (PEPFAR), and the U.S. Centers for Disease Control and Prevention (CDC), encourage the implementation of more effective data collection strategies such as Biobehavioral Surveillance (BBS) surveys, which capture essential behavioral data linked to HIV infection among these groups. These data are then used to inform policy and programming [6]. Consistent with this focus on enhanced surveillance approaches, the National Strategic Plan for HIV and AIDS Response 2010–2014 (PEN III) in Mozambique recognized the disproportionate contribution of high-risk groups to the HIV epidemic and also noted the importance of systematizing the collection of evidence about these populations in order to enhance efforts toward epidemic control [7].

Between 2011 to 2014, the Mozambican Ministry of Health (MoH), through the National Institute of Health (*Instituto Nacional de Saúde* - INS), has implemented five BBS surveys, each with the objective of collecting data on HIV prevalence, risk behaviors, access to and use of health services in these populations. The surveys focused on three key populations: female sex workers (FSW), men who have sex with men (MSM), and people who inject drugs (PWID), and two priority populations identified through consensus among key stakeholders: long-distance truck drivers (LDTD) and Mozambican workers in South African mines (MIN). Respondent-driven sampling (RDS) and time-location sampling (TLS) methodologies were used to recruit participants. A behavioral questionnaire was administered, and blood samples were collected for on-site rapid and centralized testing. The methodology and key results of each study have already been described in published reports and manuscripts. Most notably, these surveys provided the first prevalence estimates of HIV among these populations: MSM (3.7–8.2%), FSW (17.8–31.2%), PWID (19.9–50.1%), LDTD (15.4%), and MIN (22.3%) [8–17]. Table 1 summarizes the information on the implementation of the five BBS surveys among key and priority populations in Mozambique, 2011–2014.

**Table 1:** Summary information on the implementation of the first round of the Bio-Behavioral Surveillance Surveys among Key and Priority Populations in Mozambique, 2011–2014

	MSM	FSW	MIN	LDTD	PWID
**Date**	Sep-Dec 2011	Sep 2011-March 2012	Jan-May 2012	Jan-May 2012	Sep-Mar 2013–2014
**Methodology**	RDS	RDS	TLS	TLS	RDS
**Location**	Maputo Beira Nampula	Maputo Beira Nampula	Ressano Garcia, Maputo Province	Inhcope, Manica	Maputo Nampula
**Sample size**	Total: 1432 Maputo: 496 Beira: 583 Nampula/Nacala: 353	Total: 1240 Maputo: 400 Beira: 411 Nampula: 429	Total: 432	Total: 327	Total: 492 Maputo: 353 Nampula/Nacala: 139
**Inclusion criteria**	Biological male Age ≥ 18 years Had anal and/or oral sex with a biological male in the past 12 months Able to answer screening questions to verify knowledge of MSM sexual behavior In possession of a valid referral coupon Lived, worked or socialized in the survey area within the last 12 months; Ability and willingness to provide written informed consent	Biological female Age ≥ 15 years Received money in exchange for sex from someone other than a main partner in the last six months In possession of a valid referral coupon Lived, worked or socialized in the survey area within the last 6 months; Ability and willingness to provide written informed consent	Biological male Age ≥ 18 years Mozambican nationality Signed a work contract with a South African mine through TEBA Ltd. on the day of the survey Greater than one-year of experience working in a South African mine Ability and willingness to provide written informed consent	Biological male Age ≥ 18 years Truck driver who has made at least one interprovincial or international trip in the last 12 months Spent at least one night outside of their primary residence in the last 12 months Portuguese- or English-speaking Ability and willingness to provide written informed consent	Age ≥ 18 years; Injected drugs without a medical prescription within the last 12 months Ever injected drugs at any point in their lives (criterion added in December 2013) Lived, worked or socialized in the survey area within the last 12 months; In possession of a valid referral coupon Ability and willingness to provide written informed consent
**Laboratory diagnosis**	HIV Syphilis	HIV Syphilis	HIV	HIV	HIV HBV HCV
**Population size estimation**	Unique object Unique event multiplier Service multiplier Literature review	Unique object Unique event multiplier Literature review	*–*	*–*	Unique object Sequential sampling Literature review
**Implementing** **partner**	Pathfinder LAMBDA	Pathfinder PSI	Pathfinder MITRAB	Pathfinder I-TECH	Pathfinder
**Technical assistance partner**	UCSF/I-TECH CDC	UCSF/I-TECH CDC	UCSF/I-TECH CDC	UCSF/I-TECH CDC	UCSF/I-TECH CDC
**HIV Prevalence**	8.2% in Maputo (95% CI: 4.7–12.6) 9.1% in Beira (95% CI: 5.8–12.6) 3.7% in Nampula /Nacala (95% CI 1.1–7.1)	31.2% in Maputo (95% CI 24.5–37.5) 23.6% in Beira (95% CI: 18.6–29.1) 17.8% in Nampula (95% CI 13.3–22.7)	22.3% (95% CI: 17.8–26.9)	15.4% (95% CI: 11.4–19.4)	50.1% in Maputo (95% CI: 40.1–59.0) 19.9% in Nampula/Nacala (95% CI: 10.9–29.2)

The experience gained from the implementation of these surveys has provided unique opportunities to not only enhance the availability of KP-related data in the country but also to strengthen the overall HIV surveillance system in Mozambique. Lessons learned from the implementation of the BBS surveys in Mozambique, Kenya and the Caribbean have been published and focus on recruitment methodology, laboratory testing procedures, data management and analysis, and human resource selection and training [18–20]. These insights have been useful to support the implementation of BBS in low-resource settings. The objective of this paper is to examine the BBS surveys in the context of their role in strengthening the HIV surveillance system. We document the lessons learned with coordination and collaboration; workforce development and institutional capacity building; data use and dissemination; advocacy and policy impact; and financial sustainability and community impact (Table 2).

**Table 2:** Summary of lessons learned of the first round of the Bio-Behavioral Surveillance Surveys among Key and Priority Populations in Mozambique, 2011–2014

***Domain***	***Summary of lessons learned***
**Coordination & Collaboration**	• Government stewardship of survey implementation has affirmed alignment with national strategic priorities • Multisectoral collaboration enhanced ownership of survey results • Collaboration with multilateral, bilateral and research entities ensured alignment with global guidance and the use of the innovative methodological approaches • Experience of multisectoral collaboration was leveraged for policy development
**Workforce Development and Institutional Capacity Building**	• Twining model and mentorship gradually increased local capacity for survey implementation • Health informatics integrated into surveillance team to improve database development, mobile data collection tools, data visualization and survey implementation monitoring • Formalized data management practices and structures to ensure data access and use
**Data use and Dissemination**	• Increased participation in national and international conferences • Data used for global reporting and national strategic planning • Use of data by studies to for academic research (bachelor, master and PhD) • Creation of Scientific Manuscript Advisory Panel to ensure scientific integrity
**Advocacy and Policy Impact**	• Strategic information produced to monitor the HIV response • Development of Strategic Guidelines and Policy Documents.
**Financial Sustainability**	• Need to diversify funding sources to address high dependence on international donor organizations • Flexible procurement and hiring mechanisms are required to implement surveys through government system
**Community Impact**	• Enhanced visibility of KP • KP ownership of the data for advocacy purposes • Essential role of KP organizations as collaborators in the HIV response • Capacity building of KP organizations has provided opportunities for additional funding opportunities and technical collaborations

## Main text

### Lessons learned

#### Coordination & collaboration

Implementation of the BBS surveys has provided increased opportunities for multisectoral collaboration among host government entities (MoH, INS, Ministry of Labor and Provincial Health Departments), bilateral organizations (CDC, PEPFAR), multilateral agencies (UNAIDS), international academic research partners (UCSF), NGOs (ITECH, ICRH and Pathfinder International Population Services International) and civil society organizations: Association for Sexual Minority Rights in Mozambique (LAMBDA), FSW network (Tiyane Vavassate), and the National Network Against Drugs (UNIDOS). Government agencies were the stewards of the surveys, affirmed alignment with national strategic priorities, and endorsed the final results. There was gradual collaboration of government entities during the implementation process. For example, the National HIV/STI Control Program and the National Mental Health Program initially provided technical and programmatic input in the design of the surveys and eventually became principal investigators. Bilateral organizations, international academic research partner, multilateral agencies and NGOs all provided technical support, guaranteed alignment with global guidance, and the use of the latest innovative methodological approaches. In addition, the in-country NGOs provided logistical support. Civil society organizations provided contextual experience and an important link to the target populations. This participatory approach to collaboration with civil society was unprecedented in Mozambique for other national surveillance surveys and was critical for the success of the implementation of the surveys. This multi-sectoral involvement of various partners in the survey design and implementation, and dissemination of the findings has enhanced a sense of ownership of the data and set a precedence for meaningful collaboration between government agencies, technical assistance partners, and civil society organizations. For example, the multisectoral collaboration developed during the PWID study has been leveraged to support the development of a Harm Reduction Strategy drafted by the MoH-Mental Health Program and HIV/STI National Program, National AIDS Council, USAID and civil society.

The roles of the various collaborators were defined at the outset via a memorandum of understanding. The implementation of the surveys has been coordinated by a technical working group (TWG) with members from key stakeholders. The TWG is organized into sub-committees responsible for areas such as general coordination, administration and logistics, survey methods, laboratory procedures, data management, and data analysis. The TWG meets bi-weekly and regularly schedules work sessions to focus on key outputs such as protocol development, data analysis, and report writing.

#### Workforce development and institutional capacity building

One major impact of ongoing BBS survey implementation has been gradual workforce development at the INS and MoH and institutional capacity building evidenced by increasingly complex systems that support data management and use.

Technical assistance for the implementation of the BBS was initially based on a twinning model whereby technical advisors were hired by international donors and provided direct mentorship to national INS and MoH staff. To date, twinning has focused on building capacity of principal investigators, survey coordinators, data managers, and data analysts. Twinning has helped these individuals gain the knowledge and skills necessary to take on increasingly sophisticated roles of leadership within the INS and MoH over the years. As an example, the national BBS coordinator transitioned from a role of coordinating single surveys to the role of coordinator of the entire INS unit responsible for large national surveys and initiatives.

As the roles and experiences of INS and MoH staff increased and became more complex, so too did the vision of the types of skills necessary to support the implementation of high-quality surveys. For example, informatics specialists were hired to become involved in more high-tech surveillance activities that focus on providing technical assistance in database development, mobile data collection tools, data visualization and survey implementation monitoring.

The need to guarantee the proper management of BBS data coincided with a broader conversation about data management practices and structures at INS. As a result, the institution established a Data Management Unit (DMU), which manages a central data repository of all survey and surveillance data generated by INS. The DMU is staffed by data managers, statisticians, and informatics specialists, who are responsible for drafting data management and security policies, providing analysis support to various research and surveillance activities, producing reports, infographics, and supporting a website displaying summary information from key data sources. DMU staff are members of the BBS TWG and are involved in the planning, coordination, and daily implementation of the surveys.

Finally, INS implementation role has expanded over the years from governmental collaborator to primary implementor. Experience with the BBS surveys positioned INS as the key institution to lead the Mozambican arm of a multi-country study funded by the International Organization for Migration (IOM) focusing on miners and their communities [21]. Subsequent studies that have benefited from the human resources, institutional structures and experience gained through the implementation of BBS include the TB Prevalence Survey, Violence Against Children Survey (VACS), Service Availability and Readiness Assessment (SARA), and the upcoming Population-Based HIV Impact Assessment (PHIA) survey.

#### Data use and dissemination

Upon completing preliminary data analysis, the findings were shared with local stakeholders through community forums, and concise brochures developed in Portuguese and English for use during partner outreach activities. A final national report was then prepared, based upon additional data analyses and stakeholder input [8–12]. Results were also shared with other government institutions, such as Mozambique’s National AIDS Council (*Conselho Nacional de Combate ao HIV/SIDA)* and the National HIV/STI Control Program. Findings have also been disseminated at local, national, and international scientific conferences through poster and oral presentations [22–32]. Although communicating findings to the government, donors, and service delivery partners has been successful, and despite strong collaborations with central-level civil society organizations, sharing results with large numbers of key populations - especially subgroups within these key populations throughout the country (ex: high-income FSW, PWID and MSM or married MSM who may be stigmatized in their communities by their sexual behaviors) - has been a challenge due to the lack of formal networks and community organizations, especially outside of urban centers.

The data has also been used for global reporting and national target setting particularly for Global AIDS Response Progress Reporting (GARPR) and Global AIDS Monitoring (GAM), HIV prevalence and incidence estimates based on the UNAIDS-supported Spectrum package, Global Fund Proposals and PEPFAR annual plans, as well as global campaigns such as the “All In” initiative, focusing on HIV among adolescents and youth [33].

There has been a systematic approach to the use and dissemination of data for the development of abstracts for international conferences and manuscripts for international peer-reviewed journals of both INS staff and students interested in using the BBS data for their thesis and dissertation work. To date, sixteen manuscripts have been published or are under review in peer-reviewed journals [13–17, 34–44], and six students have used BBS data for their dissertations (undergraduate, masters, and doctorate). These abstracts and manuscripts have benefited from systematic and ongoing support from INS leadership and partner organizations.

To promote the analysis and dissemination of BBS scientific documents such as abstracts, manuscripts, presentations, reports, policy briefs, and brochures, the Mozambique BBS Scientific Manuscript Advisory Panel was established in 2012. This advisory panel provides oversight to ensure appropriate use of data, reviews analysis proposals to ensure scientific integrity and to avoid duplication. It was initially composed of representatives from INS, donor agencies and international research partners. Panel membership is periodically reviewed to ensure representation of the institutions leading the design and development of BBS in Mozambique. The advisory panel convenes quarterly and organizes ad hoc meetings when necessary. A data sharing policy was approved between INS and CDC in 2015, further cementing its role and institutional function. This data sharing policy was created to ensure that the data collected for BBS are used and disseminated widely and appropriately and that BBS investigators can fairly participate in the process of publishing findings, while also ensure the integrity of the use of survey data. The policy also provides guidance on data access procedures for investigators and other entities not already granted direct access to the study datasets for purposes of preparing final survey reports as described in individual BBS protocols. This data sharing policy is the first of its kind for HIV surveillance studies in Mozambique and is being used to support the development of a more generalized inter-institutional data sharing policy that encompasses the use and dissemination of various survey and surveillance activities led by INS. Unfortunately, however, the advisory panel is no longer active because it’s aims were not prioritized as result of staff turn-around of key collaborators. This highlights the importance of institutionalizing such structures.

#### Advocacy and policy impact

These BBS surveys in Mozambique produced the first HIV prevalence estimates among key populations in the country and have been used to support strategic information needs, and to identify prevention and treatment program gaps. The results of these surveys also provided the evidence necessary for key stakeholders to effectively monitor and evaluate HIV interventions and strategies directed to these populations This is exemplified by the National HIV/STI Control program’s use of BBS results to establish treatment guidelines for KPs and promote KP-friendly health provider trainings to address issues related to stigma found in the healthcare setting [45]. The National HIV program also led a recent triangulation exercise using BBS results to estimate the size of the three key population groups throughout the country, disaggregated by district. The results have been used to allocate resources, set programmatic targets and model the impact of targeted key population HIV interventions.

In addition to the use of data to identify programmatic gaps and to create the evidence base necessary for new policies, they have also been used as a powerful advocacy tool. For example, a policy brief including recommendations based on the findings from the PWID survey was produced and used by the National Mental Health Program as evidence to advocate for the introduction of harm-reduction interventions in the country. The National AIDS Council (*Conselho Nacional de Combate ao HIV/SIDA*, CNCS) is also using the data to advocate for the inclusion of key populations as part of the Prevention Roadmap.

#### Financial sustainability

The majority of funding for the implementation of the first five BBS has been through PEPFAR via CDC and their partners. Financial support has also extended to technical and implementation support which has strengthened the quality of implementation. The second round of BBS among FSW is funded by the Global Fund to Fight AIDS, Tuberculosis, and Malaria, with lab supplies purchased by CDC; UCSF continues to provide technical assistance. Unlike the collaboration with CDC during the first round of BBS, which was characterized by significant technical involvement by CDC staff and partners, the Global Fund has followed a classical donor role with limited technical involvement in the design and implementation of the surveys. This has required the BBS implementation team to learn about Global Fund policies as well as those unique to MoH in order to access funds for administrative and logistic processes. INS contributes through human resources and infrastructural support and provides general scientific oversight to ensure alignment with the objectives of the National Strategic Plan for HIV and AIDS Response. Behavioral surveillance staff also led the development of key sections of the Global Fund Proposals pertaining to research and surveillance. Despite these gains, however, the PEPFAR Sustainability Index, which monitors national investments and the sustainability landscape, highlighters that there are no domestic public or private sector funds supporting the BBS [46]. As such, there is a recognized need to diversify funding sources, however, the economic crisis in Mozambique limits the government’s ability to fund these activities at present. Finally, all future studies will include a financial evaluation to assess whether budgeted costs were met or exceeded in order to track spending and ensure the cost-effective use of limited resources.

From a practical perspective, financial structures need to be reviewed in line with the unique needs of the surveys. For example, the procurement of certain items used as “unique objects” for population size estimation – e.g., key chain flashlights, make-up kits, and hygiene kits – are difficult to acquire given the MOH/INS procurement policies which necessitate a labor-intensive and time-consuming public bidding process that requires constant communication and collaboration between the supplier and study coordinators. Hiring policies have also posed a challenge. For example, there is a need to hire a different set of staff for the formative and RDS phases of the survey who have specialized training, background, and skill sets. However, MoH considers the BBS surveys to be one unique study and, therefore, requires that the staff be the same throughout, which has had an impact on the appropriateness of short-term staff being hired. Shifts in the global economic landscape and political commitment suggest that fewer resources will be available that can be dedicated to surveillance activities, further constraining future survey implementation.

#### Community impact

The BSS surveys helped to increase the visibility of KP in Mozambique, enhanced data use for advocacy purposes, strengthened institutional capacity of KP organizations and ensured their collaboration in program implementation and strategic decision making.

Before the BBS surveys, KP were largely hidden due to stigma and discrimination. However, the results of the survey confirm the presence of these high-risk groups in Mozambique, who have taken ownership of the data and consistently use the results for advocacy. For example, the Platform for the Rights of Sex Workers network, formed after the implementation of the BSS-FSW survey, released a position paper in July 2020 where they used the results of the BBS to provide background on the situation of FSW in Mozambique and advocated for renewed commitment to human rights and social protections for sex workers in the context of the COVID-19 pandemic [47].

The KP organizations representing MSM, FSW and PWID were key collaborators in the design, implementation, analysis and dissemination of the survey findings. They received financial support to engage their peer educators in recruitment processes and provided important field oversight to implementation. As a result, these organizations have gone on to receive additional opportunities to engage in KP-specific surveys [48] and program implementation. However, the organizations still face structural challenges. For example, LAMBDA, an association for the LGBT network in Mozambique, is not yet legally recognized as a formal association and is therefore unable to receive the funding necessary to be a prime recipient of funding intended for the enhanced engagement of MSM in HIV care and treatment and therefore their contributions to the design and implementation of programming remains limited.

Finally, the meaningful engagement of KP associations is now a key element of the HIV Response.

KP organizations figure prominently in the development of the upcoming 5th National Strategic Plan for the HIV/AIDS Response (2021–2025). In addition, they are permanent members of the Country Coordinating Mechanisms of the Global Fund and are also members of the Oversight Committee charged with overseeing the programmatic and financial performance of grants financed by the Global Fund to ensure that the human and financial resources are used efficiently and effectively.

## Conclusion

Mozambique’s first experience with the design, implementation, and analysis of BBS surveys has contributed significantly to national understanding of the behavioral factors contributing to HIV transmission among key and priority populations in the country. The lessons learned provide opportunities for other limited resource settings to reflect on strategies to improve the implementation of surveys. However, the impact on strengthening the HIV surveillance system and the HIV Response should not be underestimated. The data has rendered previously hidden populations visible and resulted in enhanced capacity building, advocacy, and the development of policies and guidelines for collaboration and improved quality of care. In order to leverage the gains experienced and further systematize the lessons learned, there is a need to develop a National HIV Surveillance Plan that integrates all HIV surveillance activities under one coordinating body and emphasizes enhanced capacity of key areas, such as laboratory procedures. Such a plan must also emphasize continual human resource capacity building in the area of data analysis to support strategic planning. In addition, the importance of cultivating partnerships at the district level will guarantee the dissemination of findings to individuals who may not be connected with established key population networks and organizations. Finally, BBS surveys play an important role in providing strategic information about marginalized groups and future surveys should include other stigmatized communities, such as transgender persons, to ensure a comprehensive HIV Response. Given that traditional surveillance methodologies from routine data often do not capture hidden populations, which continue to disproportionately contribute to the HIV epidemic, it will be important to ensure that BBS surveys remain an integral element of ongoing HIV surveillance activities in Mozambique.

## Data Availability

All studies information and data sets are fully available at the Mozambique National Institute of Health (INS) data repository for researchers who meet the criteria for access to confidential data. Data are from the BBS study’s whose authors may be contacted through: www.ins.gov.mz.

## Abbreviations

BBS: Biological and behavioral survey
CDC: Centers for Disease Control and Prevention
FSW: Female sex workers
GAM: Global AIDS Monitoring
GARPR: AIDS Response Progress Reporting
HIV: Human immunodeficiency virus
ICRH: The International Centre for Reproductive Health,
INS: Instituto Nacional de Saúde (National Institute of Health)
ITECH: The International Training and Education Center for Health
KP: Key population
LAMBDA: Association for sexual minority rights in Mozambique
LDTD: Long-distance truck drivers
MIN: Mozambican workers in South African mines
MoH: Ministry of Health
MSM: Men who have sex with men
NGO: Non-governmental organization
PEN: National Strategic Plan for HIV and AIDS Response
PEPFAR: President’s Emergency Plan for AIDS Relief
PHIA: Population-based HIV impact assessment
PWID: People who inject drugs
RDS: Respondent-driven sampling
SARA: Service Availability and Readiness Assessment
STI: Sexually Transmitted Infection
TLS: Time-location sampling
TWG: Technical Working Group
UCSF: University of California in San Francisco
UNAIDS: Joint United Nations Programme on HIV/AIDS
UNIDOS: National Network Against Drugs
VACS: Violence Against Children Survey
